# Prenatal Psychosocial Distress Screening for Individuals Experiencing Pregnancies Complicated by Fetal Anomalies

**DOI:** 10.3390/jpm15070322

**Published:** 2025-07-18

**Authors:** Kara Hansen, Lisa Mische Lawson, Abigail Wilpers

**Affiliations:** 1 Department of Maternal-Fetal Medicine, Children’s Mercy Hospital, Kansas City, MO 64108, USA; 2 Occupational Therapy Education Department, University of Kansas Medical Center, Kansas City, KS 66160, USA; lmische-lawson@kumc.edu; 3 Department of Family and Community Health, University of Pennsylvania School of Nursing, Philadelphia, PA 19104, USA; awilpers@nursing.upenn.edu; 4 Research Institute, Children’s Hospital of Philadelphia, Philadelphia, PA 19104, USA

**Keywords:** fetal condition, fetal anomaly, mental health pregnancy, psychosocial fetal care, perinatal mental health, fetal care, fetal care center

## Abstract

Pregnant individuals who receive a fetal anomaly diagnosis experience significantly elevated rates of depression, anxiety, and traumatic stress—up to four to six times higher than those for individuals with low-risk pregnancies. In low-risk pregnancies, perinatal mental health conditions are the leading cause of maternal mortality and are associated with adverse birth outcomes, including preterm birth and low birth weight. These risks are likely compounded in pregnancies involving fetal anomalies due to the intersecting psychological and social burdens that complicate maternal well-being and access to care. However, there is a critical gap in understanding how these mental health symptoms translate into diagnoses, treatments, and outcomes due to the absence of a validated screening tool tailored to this population’s unique psychosocial needs. This perspective article reviews evidence, highlights the urgent need for specialized screening, and introduces ongoing research aimed at developing and validating an instrument that integrates both mental health symptoms and broader psychosocial distress. By bridging this gap, structured psychosocial screening has the potential to improve care coordination, facilitate earlier intervention, and mitigate long-term distress for individuals navigating pregnancies affected by fetal anomalies.

## 1. Introduction

Pregnant individuals receiving a fetal anomaly diagnosis experience significantly higher rates of mental health symptoms, including depression, anxiety, and traumatic stress, compared to those with low-risk pregnancies—up to four to six times more frequently. In low-risk pregnancies, mental health conditions are a leading cause of maternal mortality and are associated with adverse outcomes such as preterm birth, low birth weight, and long-term developmental challenges in children [1]. These risks are likely amplified in pregnancies involving fetal anomalies due to the intersecting psychological and social burdens that place expectant individuals at increased risk for adverse outcomes. These mental health impacts are not uniform, underscoring the need for personalized medicine approaches that consider individual psychosocial and contextual factors to guide screening and care. However, a critical gap remains in understanding how mental health symptoms translate into diagnoses, treatment, and outcomes due to the absence of a validated screening tool designed for this population’s unique psychosocial needs. This perspective article highlights the urgent need for specialized screening and introduces ongoing research aimed at developing and validating an instrument that assesses both mental health and broader psychosocial distress, ultimately improving care for this vulnerable group.

## 2. The Urgent Need for Specialized Psychosocial Assessment

Pregnancies complicated by fetal anomalies—structural, genetic, or functional abnormalities that impact fetal development—present intersecting psychological and social burdens that place expectant individuals at increased risk for adverse outcomes. These anomalies affect an estimated 3–5% of pregnancies, with many requiring specialized care, frequent medical appointments, and, in many cases, complex decision-making about fetal interventions, birth planning, abortion care, or neonatal treatment [2,3]. Many more pregnant individuals receive soft markers—suspicious but not diagnostic signs on ultrasound—requiring increased medical care throughout pregnancy [4]. Beyond the medical aspects, families often face financial strain, logistical challenges related to accessing specialized care, and shifts in social support dynamics, all of which can intensify emotional distress [5,6].

The cumulative impact of these intersecting psychological and social stressors can contribute to serious health consequences, yet research has primarily focused on individual mental health anomalies rather than the social context in which they occur. Following a fetal anomaly diagnosis, individuals can experience depression, anxiety, and traumatic stress at rates four to six times higher than those for individuals with low-risk pregnancies [7,8,9,10]. While studies have linked poor perinatal mental health to increased maternal mortality, preterm birth, and adverse child developmental outcomes, fewer have examined how social stressors—such as financial hardship, care access barriers, and disruptions to social support—may compound these risks [11,12,13]. Despite the well-documented distress experienced in this population, routine mental health screening remains inconsistent, and current perinatal screening tools fail to capture the complex psychosocial distress unique to these pregnancies [14]. A personalized medicine framework supports the development of such tools by emphasizing individualized assessment based on lived experience, emotional context, and healthcare barriers, rather than relying solely on generalized mental health instruments [15].

This perspective article highlights the urgent need for a specialized psychosocial distress screening tool and introduces ongoing research to develop an instrument that integrates both mental health symptoms and the social context in which they occur. A specialized instrument is a critical first step in psychosocial distress identification, leading to specific psychosocial interventions that support mental health. Personalized medicine aims to tailor healthcare to individuals based on their unique biological, psychological, and social characteristics, making it especially relevant for addressing the complex and varied needs of those navigating pregnancy with a fetal anomaly. By bridging the gap between standard perinatal mental health instruments and the psychosocial realities of high-risk fetal care, screening approaches grounded in personalized medicine can support earlier identification, targeted intervention, psychosocial care and referrals, and more personalized care, ultimately improving outcomes for pregnant individuals and their families [16].

## 3. Psychosocial Factors in Pregnancies with Fetal Anomalies: What We Know

### 3.1. Psychological Factors

Depression, anxiety, and traumatic stress are widespread in this pregnant population, with screening-based prevalence rates reaching up to 72% for depression, 53% for anxiety, and 39% for traumatic stress—far exceeding those seen in low-risk pregnancies [17,18,19]. The emotional toll is especially pronounced in the immediate aftermath of diagnosis, when individuals face uncertainty about the fetal prognosis, high-stakes medical decisions, and shifting expectations for their pregnancy and parenthood [9,20]. Frequent medical appointments, invasive procedures, and concerns about the quality of care contribute to heightened stress and fatigue, which may compound existing emotional distress related to the diagnosis [21,22,23]. These challenges can further exacerbate feelings of helplessness, guilt, and grief over the loss of a healthy pregnancy and baby [24,25,26]. Some evidence suggests that individuals recover from acute traumatic stress within several months, reaching levels comparable to those of pregnant individuals without a fetal anomaly [9,24,26]. Others experience stress that fluctuates but remains elevated and persists for years, making it important to identify psychological needs early. However, identification is challenging in part due to a lack of a screening instrument designed for the unique psychological experience during pregnancy with a fetal anomaly.

The uncertainty surrounding a fetal anomaly—including its pregnancy outcomes, functional implications, developmental prognosis, and family impact—is central to the experience of pregnancy with a fetal anomaly and is linked to worry, fear, and dread [27,28]. This uncertainty is also associated with poorer mental health outcomes [8,14]. Guilt and shame are common, with pregnant individuals frequently blaming themselves in the absence of cause for the anomaly [29]. Finding support in social interactions is challenging because of the social presumption of a healthy baby, frequently resulting in increased withdrawal. For example, pregnant individuals report stress in explaining the fetal diagnosis to family and especially to their other children [30,31].

In addition to ongoing uncertainty, pregnant individuals with a fetal anomaly often experience grief associated with the loss of a normal pregnancy and the birth experience and healthy baby they had imagined before receiving the diagnosis [32]. This grief can be compounded by further disruptions to their expected transition to parenthood, the emotional toll of navigating complex medical decisions during the pregnancy, and the fetal prognosis for survival [33,34,35]. The discrepancy between their anticipated experience and their current reality may contribute to feelings of isolation and emotional distress [23,36]. This profound sense of unresolved loss, combined with the ongoing emotional and logistical challenges of a high-risk pregnancy, can increase vulnerability to poor perinatal mental health.

Perinatal mental health disorders are inherently challenging to recognize for both affected individuals and healthcare professionals, leading to delayed or non-existent identification and mental health treatment [37]. This challenge is particularly pronounced in pregnancies complicated by a fetal anomaly, where medical care is primarily focused on the anticipated baby, and pregnant individuals frequently neglect their own well-being [32]. Once identified, integrated psychosocial support and mental health treatment can support positive outcomes for pregnant individuals. While screening is essential for identifying psychological support needs and is both feasible and desired in perinatal healthcare settings, existing measures fail to capture the complex psychosocial challenges faced by this population [38,39].

Failure to address these mental health needs can have serious consequences for both maternal and infant health. Untreated perinatal mental health anomalies are associated with higher rates of suicide, substance use, preterm birth, low birth weight, and impaired parent–child bonding [11,40,41,42]. Moreover, unidentified and untreated mental health needs often persist for years following childbirth [42]. Despite these risks, mental health support within fetal care settings remains fragmented and inconsistently integrated into routine care [32]. As a result, many individuals navigate the complex emotional landscape of a fetal anomaly diagnosis without adequate psychosocial resources, potentially contributing to long-term psychological distress.

### 3.2. Social Factors

Preliminary evidence suggests a connection between social and psychological factors in pregnancies complicated by fetal anomalies. Among the limited studies examining these factors together, financial hardship, transportation barriers, and food insecurity have been associated with higher rates of depression, anxiety, and reduced empowerment [7,43]. Additionally, patients on state insurance have been found more likely to screen positive for clinically significant mental health symptoms [43,44,45]. While these findings reinforce the link between social factors and perinatal mental health, they do not explain how these influences interact with the unique experiences of individuals navigating a fetal anomaly diagnosis. Understanding the drivers of these associations remains a critical gap in the evidence. The following section examines how some social factors intersect with care access, use, and outcomes and may contribute to psychological distress.

Geographic and logistical barriers significantly impact access to timely and accurate fetal diagnoses, shaping both medical and psychosocial outcomes. Access to high-quality prenatal imaging, experienced sonographers, and maternal–fetal medicine specialists varies widely by region, leading to unequal detection rates for fetal anomalies [46,47]. Accessing abortion care following a fetal anomaly diagnosis is challenging due to legal limits in many states, creating limited access and increased travel. Individuals in rural areas or regions with limited diagnostic services are more likely to experience delayed or missed diagnoses, particularly for anomalies requiring specialized imaging, such as congenital heart defects and neural tube defects [48,49]. These delays have serious consequences, as some fetal anomalies have time-sensitive intervention windows, and late detection may preclude eligibility for treatments like maternal–fetal surgery [50]. While these in utero procedures can improve neonatal outcomes, successful intervention depends on early identification and timely referral to specialized fetal care centers [51].

Accessing care from a fetal care center provides more than just medical interventions; centers provide interdisciplinary care and counseling, and a number of fetal care centers also offer essential psychosocial support, which may be less accessible in standard obstetric settings [39]. These centers coordinate psychosocial care services, resource navigation, and peer support, helping individuals process grief, access financial and logistical assistance for care, and find community in navigating the loss of a typical pregnancy experience, though significant variability among centers exists [52,53]. However, many fetal care centers are concentrated in regional hubs, requiring extensive travel, temporary relocation, or reliance on inconsistent local healthcare professionals for follow-up care [7]. These barriers not only limit access to medical interventions but also reduce opportunities for individuals to receive comprehensive psychosocial support. Growing evidence supports that being unable to access the desired perinatal care, regardless of the specific intervention sought, is associated with increased distress, anxiety, and lower self-esteem [54,55,56].

Once a fetal anomaly is diagnosed, individuals are likely to face significant financial strain, regardless of the care pathway they choose. Perinatal care—whether it involves abortion or ongoing fetal monitoring, in utero treatment, neonatal interventions, or palliative care—often requires out-of-state travel, extended time off work, and significant out-of-pocket expenses. A study of 453 fetal care patients found that 38.2% of families reported moderate to severe financial burdens, with nearly 10% spending over 5000 USD on non-medical costs alone [7]. These financial pressures can make it difficult to afford mental health support when it is not coordinated into their care and may force individuals to prioritize working to maintain income and health insurance over engaging in self-care. At the same time, existing social relationships are often disrupted following a diagnosis, as many individuals withdraw due to stigma, grief, or isolation, while others experience strained relationships with partners or family members who struggle to provide emotional support [57,58]. Many patients report feeling uncertain about where to turn for mental health support, as their distress is often overshadowed by the medical management of the fetus [59,60].

## 4. Bridging the Psychosocial Gap

Despite the well-documented mental health burden and social stressors associated with fetal anomalies, there is no standardized approach for assessing or addressing psychosocial distress in this population. Some fetal care centers assess mental health needs using a combination of multiple validated instruments, including general psychiatric tools such as the Patient Health Questionnaire-9 and the Impact of Events Scale, alongside perinatal-specific scales like the Edinburgh Postnatal Depression Scale, the Perinatal Anxiety Screening Scale, and the Postpartum Depression Screening Scale [5,8,61]. However, these instruments have not been validated for the fetal anomaly population and fail to assess diagnosis-specific grief, medical decision-making distress, or the structural barriers that shape mental health outcomes. In addition, the use of multiple overlapping instruments may introduce redundancies, particularly among the overlapping and intersecting constructs contained in traditional depression, anxiety, and traumatic stress measures, thereby increasing patient burden and response fatigue and potentially leading to incomplete or inaccurate assessments [62,63]. Debate persists among perinatal mental health professionals regarding whether the anxiety component in depression screens is sufficient or if a separate anxiety screening is necessary [64,65]. Without a validated framework that integrates both mental health symptoms and broader psychosocial stressors, healthcare professionals may miss key distress indicators, leaving individuals unidentified and without necessary support.

To address this gap, research is underway to develop and validate a Psychosocial Distress with Fetal Anomaly Scale (PD-FAS)—a screening instrument specifically designed to assess the emotional, social, and structural stressors that shape distress in this population. Instrument development is a multi-step, iterative process that requires both conceptualizing the construct and evaluating item relevance through expert assessment [66,67]. By supporting early identification and linking individuals to targeted psychosocial interventions, the PD-FAS contributes to a personalized medicine approach—one that aligns care with each individual’s unique experiences, risks, and needs [68]. The Delphi method guided the initial phase of this study, as it provides a structured, mixed-methods approach to achieving expert consensus and establishing content relevance [69,70]. This method is particularly valuable in contexts where existing evidence is scarce or inconsistent, allowing for the systematic refinement of instrument domains based on interdisciplinary expertise [71]. Through iterative rounds of expert review, the Delphi process helps ensure that the instrument’s content reflects real-world clinical challenges, reducing potential item bias and increasing applicability across diverse care settings [72].

This research applies the Delphi methodology within a multidisciplinary framework, incorporating expertise from maternal–fetal medicine, psychology, social work, and individuals with lived experience of fetal anomaly to ensure comprehensive representation of psychosocial distress in fetal care settings. By integrating perspectives from both clinicians and patients, the PD-FAS aims to capture the complexities of distress beyond what existing perinatal mental health measures assess. A key strength of this research is its foundation in established methodologies that previously led to the development and validation of a person-centered, patient-facing instrument for the fetal care population [73,74]. By employing similar rigorous methodological approaches, this research centers the pregnant patient, draws from validated measures to tailor content to this population, and engages interdisciplinary experts to capture the intersections of clinical care, social contexts, and mental health implications.

## 5. Future Research and Implications for Clinical Practice and Policy

With the Delphi process nearing completion, the next phase of research will focus on establishing the psychometric properties of the PD-FAS through rigorous validation methods. Piloting the instrument in diverse fetal care settings will allow for an exploratory factor analysis and a confirmatory factor analysis to assess its construct validity and refine the item structure. Additional testing will evaluate reliability measures, including internal consistency and test–retest reliability, to ensure the scale performs consistently over time. Criterion validity testing will compare the PD-FAS to existing validated measures to determine its effectiveness in identifying psychosocial distress within this population. Multi-site studies will be critical to examining its generalizability across different healthcare settings, ensuring that the instrument is applicable and feasible in a range of clinical environments.

Once validated, the PD-FAS has the potential to improve clinical care by providing a structured, evidence-based approach to psychosocial distress screening in perinatal settings. Implementing and standardizing a screening tool that accounts for these intersecting burdens of psychosocial and social stressors would facilitate earlier identification of at-risk individuals, improved care coordination, and greater recognition of social stressors that impact both maternal and fetal outcomes. In addition to using a screening instrument tailored to the population they serve, fetal care centers should embed psychosocial professionals (e.g., social workers, mental health clinicians, psychologists, and/or psychiatrists) into their clinical teams to ensure timely access to care, while reducing stigma and enabling early identification and intervention for psychosocial distress [32]. While evidence-based interventions specific to pregnancies complicated by fetal anomaly are limited, approaches used in broader perinatal and medically complex populations—such as cognitive behavioral therapy, interpersonal therapy, behavioral activation, and resilience-building—show promise [11,28,45]. Low-intensity formats, including guided self-help and digital interventions, may offer scalable, accessible, and cost-effective options [11,28].

Establishing infrastructure for integrated psychosocial care requires investment in staffing, training, and workflows but yields significant clinical and economic benefits. A validated, tailored screening instrument is a critical first step to identifying individuals experiencing distress and matching them with appropriate levels of intervention. Proactively addressing psychosocial needs during pregnancy enhances maternal mental health, strengthens parent–infant bonding, and improves long-term child outcomes. In contrast, the cost of inaction—ranging from untreated perinatal mood and anxiety disorders to increased healthcare utilization—far exceeds the investment required to build comprehensive psychosocial support into fetal care settings [32].

Additionally, integrating the PD-FAS into routine care could enhance patient–clinician communication, offering a structured way to assess and discuss mental health concerns and social stressors, including emotional burden, interpersonal distress, grief, and healthcare-related distress. By capturing both psychological symptoms and structural barriers, this tool will help healthcare professionals deliver more integrated, person-centered care that acknowledges the full scope of challenges faced by individuals navigating pregnancies complicated by fetal anomalies. Incorporating personalized medicine into psychosocial screening could further optimize and tailor care to each individual’s unique emotional, social, and medical needs.

## 6. Conclusions

While further validation is needed before policy applications can be considered, this research establishes a foundation for future recommendations on incorporating routine psychosocial distress screening into high-risk perinatal care. As evidence grows supporting the effectiveness of structured psychosocial screening in this population, findings from this work could inform best practices and guidelines for ensuring that psychosocial distress is recognized and addressed as a standard component of maternal fetal medicine. By prioritizing the integration of psychosocial distress into routine fetal care, healthcare systems can move toward a more comprehensive and patient-centered approach that acknowledges the medical, social, and emotional complexities of these pregnancies and focuses on early identification and intervention. Ultimately, identifying and addressing the psychosocial needs of individuals during pregnancy with a fetal anomaly is a critical step toward advancing equity and holistic care in perinatal medicine.

## Data Availability

No new data were created or analyzed in this study. Data sharing is not applicable to this article.

## Abbreviation

The following abbreviation is used in this manuscript:

PD-FAS	Psychosocial Distress with Fetal Anomaly Scale
